# Effects of Body Posture on Voice Range Profile Performance in Untrained Vocally Healthy Individuals

**DOI:** 10.1111/1460-6984.70130

**Published:** 2025-09-23

**Authors:** Ben Barsties v. Latoszek, Pia Droßard, Ferdinand Binkofski, Ewa v. Latoszek

**Affiliations:** ^1^ School of Health, Education and Social Sciences SRH University of Applied Sciences Heidelberg Düsseldorf Germany; ^2^ Division of Clinical Cognitive Sciences Medical Faculty of the RWTH Aachen University Aachen Germany; ^3^ Studio for professional voice education and speech culture Cologne Germany

**Keywords:** acoustic analysis, posture, vocal function, voice assessment, voice disorders, voice range profile, VRP

## Abstract

**Background:**

The voice range profile (VRP) is an acoustic measurement in vocal function voice assessment. While several factors influencing VRP outcomes are known, the impact of body posture during VRP recordings remains unexplored.

**Aims:**

To investigate the effects of standing and sitting posture on VRP performance in vocally healthy individuals.

**Methods and Procedures:**

Thirty vocally healthy and untrained participants were randomised into two groups. Group 1 performed VRP measurements first in a sitting position, followed by standing. Group 2 completed the tasks in reverse order. VRP parameters were compared between sitting and standing positions, and training effects between the first and second measurements were analysed.

**Outcomes and Results:**

No significant differences were found in any VRP parameter between sitting and standing positions (all *p* values > 0.05), with nearly all effect sizes being very small or small. Furthermore, no training effect was observed among the two trials in each group (all *p* values > 0.05, ICC > 0.75, very small or small effect sizes).

**Conclusions and Implications:**

Posture (sitting vs. standing) did not significantly influence VRP performance in the first instance when considering people without voice disorders. This might suggest flexibility in patient positioning during VRP assessments, potentially simplifying clinical protocols without compromising data integrity.

**WHAT THIS PAPER ADDS:**

*What is already known on this subject*

- Voice Range Profile (VRP) is a standard acoustic tool for assessing vocal function influenced by several factors such as recording methods, software, and patient characteristics. Though practical guidelines often recommend performing VRP assessments in a standing position, no empirical evidence has previously evaluated the effect of body posture on VRP outcomes. Thus, the clinical necessity of specific postural instructions during VRP remains unclear.

*What this paper adds to the existing knowledge*

- This study is the first to investigate how body posture (sitting vs. standing) affects VRP performance in vocally healthy and untrained individuals. Using a randomised crossover design, it found no significant differences in any VRP parameter between sitting and standing conditions. Additionally, there was no observed training effect between repeated measurements, suggesting high test‐retest reliability. These findings challenge the assumption that standing is superior for VRP assessments.

*What are the potential or actual clinical implications for this work?*

- The results suggest that body posture may not substantially influence VRP outcomes in vocally healthy and untrained individuals. Therefore, conducting VRP assessments in either sitting or standing positions could be considered in certain clinical contexts, especially when accommodating patient needs. However, given the study's focus on healthy participants, these findings should be interpreted with caution. Further research involving diverse populations, including individuals with voice disorders or specific postural conditions, is needed before recommending broader changes to clinical protocols.

## 1 Introduction

Objective acoustic methods are widely used in voice diagnostics to assess voice disorders (Roy et al. 2013). This assessment method is influenced by a range of factors that must be carefully considered, including minimum hardware specifications, algorithmic variations across software, room acoustics, the impact of face masks in accordance with hygiene standards, as well as the vocal behaviour and performance conditions during recording of the individuals being assessed (Barsties v. Latoszek et al. 2025). The voice range profile (VRP) is a prominent acoustic measurement that graphically represents vocal capabilities by plotting fundamental frequency (F0) against intensity (Ternström et al. 2016). The VRP displays the range of frequencies, measured in Hertz (Hz) or semitones (ST), on the horizontal axis and corresponding sound intensities, measured in decibels (dB), on the vertical axis (Cutchin et al. 2020).

**FIGURE 1:**
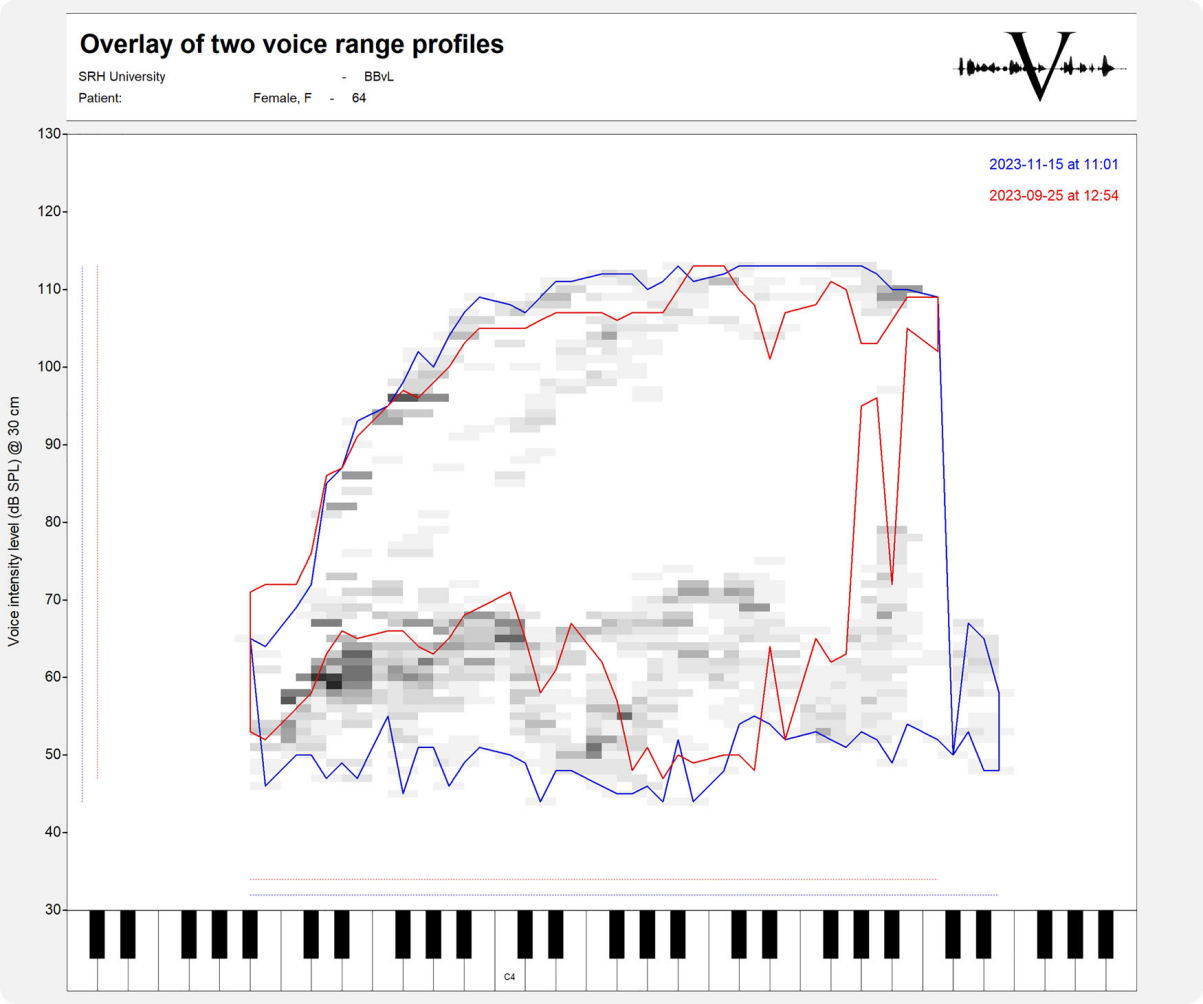
Example of a VRP in Vox Phonetography (Nijkamp 2022) for sustained phonation of the vowel [a] by a 64‐year‐old woman diagnosed with functional dysphonia. The assessments were conducted before and after a 4‐week voice treatment program. Two graphs illustrate the VRP boundaries: the pre‐treatment profile is delineated by a red line, while the post‐treatment profile is indicated by a blue line. For comprehensive details, refer to the Vox Phonetography manual.

The VRP exhibits a characteristic shape despite inter‐individual variability influenced by factors such as different recording methods (manual vs. automatic), warm‐up exercises, vowel choice, trained vocal status, gender, and age (Titze 1992a; Coleman 1993; Heylen et al. 1996; Siupsinskiene 2003; Ternström et al. 2016; Berger et al. 2022). Normal fluctuations between consecutive VRP measurements are approximately 2 dB for intensity and 3.5% for frequency (Printz et al. 2018). Figure 1 shows two VRP's plotted on top of each other of a voice patient who has exceeded the tolerance fluctuations at certain frequencies and loudnesses (before therapy boundaries line in red and after therapy boundaries line in blue). In general, a larger VRP indicates better vocal function, reflecting the balance between vocal capacities and the demands placed on the voice (Coleman 1993). From a clinical perspective, the VRP serves as an objective measure of vocal function, enabling the assessment of normal ranges for individuals with and without voice disorders, investigation of vocal changes due to vocal stress or treatment, determination of pitch range and intensity limits, and evaluation of register transitions (Heylen et al. 1996; Ikeda et al. 1999).

The VRP also provides insights into individual phonation characteristics by analysing frequency and intensity limits as well as the shape and quality of its single cells. The frequency range encompasses both modal (chest voice) and falsetto (head voice) registers or entire vocal genres in classical singing (e.g., soprano, mezzo‐soprano, alto, tenor, baritone, bass). Typically, only the highest and lowest tones are interpreted clinically. The lowest tone corresponds to relaxed and shortened vocal folds with moderate transglottal air pressure, while the highest tone involves elongated and tense vocal folds driven by cricothyroid muscle activity combined with high subglottic pressure (Ternström et al. 2016). The boundaries of the VRP define upper and lower loudness limits across the frequency range. The lower boundary reflects phonation thresholds that depend on vocal fold elasticity and flexibility required for soft phonation (Titze 1992b). Accurate determination of this threshold requires both auditory and visual evaluation by examiners to ensure patients achieve their quietest intensity with controlled phonation attempts. The upper boundary of the VRP represents maximum vocal intensity, influenced by factors such as glottal airflow, mouth opening, vocal tract acoustics, and register transitions. It reflects the vocal effort required to produce loud phonation, involving increased tension in laryngeal muscles and elevated subglottic pressure (Ternström et al. 2016). Notable features in VRP boundaries include a depression marking the transition between modal and falsetto voices, and relatively stable loudness levels at low‐to‐middle pitches (Ternström et al. 2016). However, the physiological limitation of producing very loud voices at low pitches often leads individuals to raise pitch for increased intensity. Compensatory pitch adjustments to achieve higher vocal intensity can lead to vocal fatigue or discomfort over time (Solomon 2008).

The slope of the VRP typically increases by approximately 8–10 dB per octave with rising loudness levels (Titze and Sundberg 1992), reflecting reduced loudness at low frequencies compared to softer voices at higher frequencies (Ternström et al. 2016). Beyond its boundaries, advanced acoustic parameters such as jitter, shimmer, harmonics‐to‐noise ratio, singing power ratio, crest factor, and cepstral peak prominence, smoothed, can be incorporated into VRP analyses to investigate phonation strategies across individual cells within the profile (Pabon 1991; Pabon and Ternström 2020; Nijkamp 2022). These parameters enhance understanding of phonatory mechanisms and abnormalities in vocal sound production.

In summary, the VRP is an indispensable tool in voice diagnostics due to its ability to provide quantitative assessments of vocal function across diverse acoustic dimensions. Its applications extend from clinical evaluations of voice disorders to research exploring phonation strategies and treatment outcomes. By integrating traditional boundaries with advanced acoustic analyses, the VRP offers a comprehensive framework for assessing vocal performance.

While several factors influencing VRP characteristics have been identified, as mentioned earlier, the impact of body posture (sitting vs. standing) as performance condition during recording remains largely unexplored. Given the partly clinical recommendation to perform VRP assessments in a standing position (Berger et al. 2022), yet without a clear rationale, the present study aimed to investigate the potential influence of posture on VRP performance. Furthermore, as repeated VRP measurements may elicit a training effect on vocal function, this study also examined the presence and magnitude of such an effect. A comprehensive understanding of these controllable factors is crucial for ensuring the reliability and validity of VRP assessments.

## 2 Methods

The study was conducted in accordance with the Declaration of Helsinki and approved by the Ethics Committee (see Ethics Statement).

### 2.1 Participants

A total of 30 volunteers were recruited from the vicinity of SRH University, Campus Düsseldorf, Germany, to participate in this study. The sample comprised 12 male and 18 female participants. Participant ages ranged from 18 to 58 years, with a mean age of 32.3 years. Inclusion criteria mandated that all participants be native German speakers, vocally healthy, and untrained. Vocal health status was determined using the German versions of the Voice Handicap Index (VHI) and the Vocal Fatigue Index (VFI). Participants were required to have a VHI total score of 14 or less, a VFI score of cluster 1 below 18 and cluster 2 scores above 8 to be classified as having normal voice status (Nawka et al. 2003; Barsties v. Latoszek et al. 2023). Participants were exclusively untrained non‐professional voice users. This untrained status was additionally defined by the absence of any formal vocal education or structured voice‐related activities, including but not limited to choir participation, singing lessons, or theatrical performance experience. Exclusion criteria included the presence of any voice disorder within the 12 months preceding the study, previous voice therapy, prior experience with voice range profile recordings, odynophonia, habitually low vocal loading, the presence of body prostheses that would affect upright stance, any difficulty maintaining a standing or seated position during the examination period, or reliance on a wheelchair.

### 2.2 Equipment and Recording Protocol

Voice samples were recorded using a head‐mounted AKG C544l condenser microphone coupled with an AKG MPA V L adapter, adhering to established recommendations for hardware standards in acoustic voice and speech analysis (Patel et al. 2018). All recordings were digitised in WAV format through a Focusrite iTrack Solo audio interface. Vox Phonetography software was used for both data acquisition and processing of the voice range profile.

Prior to each participant recording session, the Vox Phonetography system underwent calibration (Nijkamp 2022) following the established procedure described by Maryn and Zarowski (2015). Intensity calibration was performed using a calibrated PCE‐322A sound‐level meter (PCE Instruments, Meschede, Germany) with IEC 61672‐1 Type‐II classification, providing ±1.4 dB tolerance in measurement accuracy. To ensure optimal recording quality, all voice samples were collected in a quiet environment where ambient noise remained consistently below 50 dB(C) (Titze 1995).

Vox Phonetography is a tool designed for the automatic measurement of voice range profiles (Nijkamp 2022). It provides measurements and graphical representations of both intensity and fundamental frequency. The evaluation yielded key limit values including the highest fundamental frequency (F0‐high), the lowest fundamental frequency (F0‐low), maximum intensity (I‐max), and minimum intensity (I‐min). From these values, the semitone range and intensity range are derived, which collectively define the characteristics of an individual's VRP.

### 2.3 Study Procedure

The study protocol for the voice range profile measurement of the chest and head voices on the sustained vowel /a/ was conducted using the Vox Phonetography software following standardised instructions by Barsties v. Latoszek et al. (2025), and recommendations by Cutchin et al. (2020). Each VRP measurement lasted approximately 10 min per participant. The procedure began with the assessment of the lower boundary of the vocal range, measured from low to high using glissando techniques, such as imagination aids like soft yawning. If necessary, discrete half‐step tones were applied to achieve maximum performance of the total range. These half‐step tones were played before by the software's integrated piano and controlled by the examiner. Multiple attempts were allowed to measure the maximum performance of the semitone range at a low volume. Subsequently, the upper boundary of the vocal range was also determined, progressing from low to high using techniques similar to those applied for the lower boundary. For this boundary, participants made fewer attempts at maximum intensity to minimise potential signs of vocal fatigue and related vocal complaints. To ensure accurate performance during VRP measurements, the examiner demonstrated each exercise as a model for participants to follow. In instances where vocal deviations occurred during recording, overtones, vocal fry registrations, and individual recordings deviating by more than 4 dB or 4 semitones from the connected range of the VRP were excluded from analysis in accordance with Hallin et al. (2012).

The following acoustic parameters from the VRP have been extracted: F0‐high, F0‐low, I‐max, I‐min, the intensity range in dB, and semitone range in ST.

A cross‐over study design was implemented to investigate the effects of body posture on VRP results in accordance with the principles outlined by Kenward and Jones (2007). Each participant completed two VRP measurements sequentially: one in a sitting posture and one in a standing posture. Participants were randomised into two groups using permuted block randomisation generated with Excel software to minimise bias. Group 1 followed an AB sequence, performing the VRP measurement in the sitting posture first followed by the standing posture. Group 2 followed a BA sequence beginning with the standing posture and then transitioning to the sitting posture. This cross‐over design allowed each participant to serve as their own control, enabling within‐subject comparisons of VRP parameters between sitting and standing postures while reducing variability caused by inter‐subject differences. To address potential carry‐over effects between the first and second VRP measurements, statistical analyses were conducted to assess whether exposure to the first measurement influenced performance during the second measurement. Specifically, this evaluation aimed to identify any training effects or biases that could confound the results. By accounting for these potential effects, this study ensured that observed differences in VRP parameters were attributable to body posture rather than order effects or practice‐related improvements.

### 2.4 Statistical Analysis

Statistical analyses were performed using IBM SPSS Statistics for Windows Version 28.0 (IBM Corp, Armonk, NY). The normality of the distribution of VRP parameter outcomes for each trial was evaluated using the Shapiro–Wilk test. A *p* value greater than 0.05 was considered indicative of a normal distribution.

To examine the effect of body posture on VRP outcomes, either the Wilcoxon signed‐rank test or the paired *t*‐test was applied, depending on the results of the normality test. Furthermore, Cohen's *d* was calculated to assess the effect size of VRP‐related differences in body posture. The training effect between the first and second VRP performances was evaluated using the intraclass correlation coefficient (ICC), with additional analysis conducted using either the Wilcoxon signed‐rank test or the paired *t*‐test, as determined by the normality of the data. Cohen's *d* was again evaluated to assess the effect size of a potential training effect. The ICC is a reliability coefficient ranging from 0.00 (no reliability) to 1.00 (perfect reliability). While no absolute thresholds exist for interpreting ICC values, a general guideline suggests that values above 0.75 indicate good reliability, whereas values below 0.75 suggest poor to moderate reliability (Portney and Watkins 2000).

Cohen's *d* values from the paired *t*‐test were automatically generated by SPSS, whereas the results of the *Z*‐values from the Wilcoxon test were first manually converted into a correlation and then transformed into Cohen's *d* (Cooper et al. 2009). Effect sizes are defined as small at *d* = 0.2, medium at *d* = 0.5, and large at *d* = 0.8 (Cohen 1992). A *p* value of < 0.05 was considered statistically significant.

## 3 Results

The results of the normality test indicated a normal distribution for I‐max, intensity range, and semitone range (all *p* values > 0.05). In contrast, I‐min, F0‐high, and F0‐low exhibited a non‐normal distribution (all *p*‐values < 0.05).

Table 1 presents the descriptive statistics of VRP performances across females (*n* = 18) and males (*n* = 12) in sitting and standing postures. To evaluate the effect of body posture on VRP outcomes independent of gender (*n* = 30), a statistical analysis based on mean differences in the quantitative VRP parameters was performed (see Table 2). The results demonstrated no statistically significant effect of body posture on any VRP measure. The effect sizes were nearly all very small or small, with only I‐max yielding a medium effect size for a minor mean difference of 0.87 dB. Furthermore, no training effect was observed when comparing two consecutive VRP executions in different body postures (see Table 3). The ICC analysis confirmed sufficient reliability for all parameters between the first and second measurements (all ICC values > 0.75). No statistically significant differences were observed between these time points (all *p* values > 0.05), and the effect sizes were very small or small (i.e., I‐max).

**TABLE 1:** VRP results among sitting and standing body posture separated between males and females.

		Sitting		Standing	
Gender (number)	VRP‐parameter	Mean	SD	Mean	SD
Female (18)	I‐max	101.17 dB	5.66 dB	101.28 dB	5.84 dB
	I‐min	45.28 dB	3.97 dB	45.00 dB	3.82 dB
	Intensity range	55.89 dB	7.30 dB	56.22 dB	7.55 dB
	F0‐high	795.94 Hz	106.65 Hz	791.22 Hz	102.69 Hz
	F0‐low	158.06 Hz	14.06 Hz	158.50 Hz	12.58 Hz
	Semitone range	27.89 ST	3.03 ST	27.72 ST	2.74 ST
Male (12)	I‐max	102.17 dB	3.01 dB	99.83 dB	2.76 dB
	I‐min	43.67 dB	2.15 dB	43.25 dB	1.82 dB
	Intensity range	58.50 dB	2.28 dB	56.58 dB	3.23 dB
	F0‐high	519.50 Hz	77.10 Hz	519.67 Hz	73.86 Hz
	F0‐low	80.00 Hz	13.15 Hz	79.75 Hz	11.54 Hz
	Semitone range	32.42 ST	3.45 ST	32.50 ST	3.06 ST

Abbreviations: dB, decibel; F0‐high, highest fundamental frequency in voice range profile; F0‐low, lowest fundamental frequency in voice range profile; Hz, Hertz; I‐max, maximum intensity in voice range profile; I‐min, minimum intensity in voice range profile; SD, standard deviation; ST, semitones; VRP, voice range profile.

**TABLE 2:** Effect of body posture performance of all participants (*n* = 30) for the various VRP parameters.

	Sitting vs. Standing			
VRP parameter	Mean differences	SD	*p* Value	Cohen's *d*
I‐max	+0.87 dB	3.31 dB	0.152^*^	0.542^a^
I‐min	+0.33 dB	2.96 dB	0.543^**^	0.112
Intensity range	+0.57 dB	4.56 dB	0.385^*^	0.321^b^
F0‐high	+2.76 Hz	14.59 Hz	0.308^**^	0.190
F0‐low	−0.17 Hz	5.43 Hz	0.868^**^	0.031
Semitone range	+0.07 ST	0.94 ST	0.806^*^	0.090

Abbreviations: dB, decibel; F0‐high, highest fundamental frequency in voice range profile; F0‐low, lowest fundamental frequency in voice range profile; Hz, Hertz; I‐max, maximum intensity in voice range profile; I‐min, minimum intensity in voice range profile; SD, standard deviation; ST, semitones; VRP, voice range profile.

^a^ medium effect size.

^b^ small effect size.

^*^Wilcoxon sign ranked test.

^**^paired sample *t*‐test.

**TABLE 3:** Reliability results between the first and second VRP measurement.

VRP parameter	ICC	*p* Value	Cohen's *d*
I‐max	0.854	0.262^*^	0.419^a^
I‐min	0.756	0.393^**^	0.158
Intensity range	0.830	0.682^*^	0.150
F0‐high	0.998	0.308^**^	0.190
F0‐low	0.996	0.973^**^	0.006
Semitone range	0.984	0.854^*^	0.067

Abbreviations: F0‐high, highest fundamental frequency in voice range profile; F0‐low, lowest fundamental frequency in voice range profile; I‐max, maximum intensity in voice range profile; I‐min, minimum intensity in voice range profile; VRP, voice range profile.

^a^ small effect size.

^*^Wilcoxon sign ranked test.

^**^paired sample *t*‐test.

## 4 Discussion

The present study aimed to evaluate the effect of body posture on voice range profile performance in untrained vocally healthy volunteers. Using a cross‐over study design, we measured single quantitative frequency and intensity boundary parameters, as well as their respective ranges, in two different body postures: sitting and standing. The findings revealed no significant differences in VRP performance between these two postures (all *p* values > 0.05), with effect sizes that were practically negligible. Furthermore, the observed ranges of mean differences were within the tolerance limits of automatic VRP recordings (Printz et al. 2018). Additionally, no training effect was detected between the two time points of measurement (ICC > 0.75, all *p* values > 0.05, very small or small effect sizes).

Although a recent systematic review on methodological aspects of VRP performance did not address body posture (Cutchin et al. 2020), some clinical recommendations suggest that VRP assessments should be performed in a standing position (Berger et al. 2022). To our knowledge, this is the first study which investigated the clinical effects of body posture on VRP performance. The present results confirmed no rationale to prescribe a body posture for VRP assessments. Previous research on other vocal function outcomes has also reported no significant differences for males and females between sitting and standing postures regarding fundamental frequency, intensity, maximum phonation time, subglottic pressure at comfortable phonation, electroglottographic contact quotient, glottal airflow rate during phonation, glottal aerodynamic resistance, glottal aerodynamic efficiency, and self‐perceived phonatory effort (Gilman and Johns 2017; Gong et al. 2024). The previous studies included participants with characteristics comparable to those in the present study. To minimise potential confounding variables, such as learned techniques or compensatory mechanisms, all participants in these studies were vocally healthy individuals with no prior vocal training. This methodological approach established a neutral and controlled baseline for evaluating posture‐related effects on voice.

However, our results do not contradict the broader understanding that incorrect or misaligned postures of the head and neck region may negatively impact vocal performance. Misalignments in posture of the head‐neck region have been shown to negatively affect vocal function by altering the biomechanical efficiency of the phonatory system. For instance, forward head posture has been associated with increased tension in the extrinsic laryngeal muscles and reduced vocal efficiency due to imbalances in cervical spine alignment (Wilson Arboleda and Frederick, 2008; Gilman and Johns 2017). Also, changes of the configuration of the oropharynx and hypopharynx in different head positions could be observed, which might have an impact on vocal tract volume, and, thus, on the voice sound (Schade et al. 2004). Current understanding of how misalignments in posture of the head‐neck region influence VRP performance is limited, underscoring the necessity for continued investigation. Nevertheless, misalignments of the entire standing body or standing on an unstable surface have not been shown to induce significant changes in voice aerodynamic parameters or in the activation levels of muscles involved in the phonation‐breathing process, even among vocally healthy individuals with singing experience (Castillo‐Allendes et al. 2025).

### 4.1 Limitations and Future Direction

The present study has some limitations that should be acknowledged, as they may not only impact the generalizability of the findings but also inform directions for future research.

First, the study sample comprised only untrained vocally healthy adults within young and middle‐aged groups. To enhance the applicability of these findings, future research should extend investigations to trained voice users, individuals with voice disorders and/or postural disorders of the head‐neck region, children, and older adults. This broader approach would allow for a more comprehensive understanding of the potential influence of body posture on VRP performance.

Second, in crossover study designs, ensuring an adequate washout period between conditions is essential. In the present study, the vocal load induced by the two consecutive VRP measurements was relatively low, consistent with the VRP procedure protocol of Barsties v. Latoszek et al. (2025), and post hoc analyses revealed no significant training effects between sessions. Nonetheless, the short interval between the two measurements may have allowed participants to recall or imitate aspects of their earlier performance, rather than responding fully independently. Extending the intersession interval, for example by implementing several days of separation as suggested by Berger et al. (2022), could reduce such residual influences and improve the reliability of the findings.

A third limitation is the absence of laryngeal imaging techniques such as laryngoscopy, laryngostroboscopy, videokymography, or high‐speed digital imaging. The lack of these assessments precludes definitive conclusions regarding the structural and functional status of the vocal folds and surrounding laryngeal structures. However, the VHI and the VFI serve as effective screening tools for identifying voice disorders (Behlau et al. 2016; Barsties v. Latoszek et al. 2023; de Oliveira Lemos et al. 2025). In addition, the mean results of the VRP parameters listed in Table 1 are aligned with normative performance thresholds (Friedrich and Dejonckere 2005). Nevertheless, the robustness of the findings would be enhanced by additional objective assessments of laryngeal structure and function. Future research should integrate endoscopic imaging techniques to provide a more comprehensive understanding of vocal physiology and strengthen the interpretation of VRP findings.

## 5 Conclusion

This study investigated the impact of body posture on voice range profile performance in untrained vocally healthy individuals. The findings indicate no significant differences in VRP measures between sitting and standing postures, nor was a training effect observed. These results suggest that body posture may not influence VRP outcomes, supporting the feasibility of conducting VRP assessments in either posture without compromising measurement reliability. Further research could explore whether these findings apply to trained voice users, other age groups, or individuals with voice disorders.

## Ethics Statement

The study was conducted in accordance with the Declaration of Helsinki and approved by the Ethics Committee of the German Academic Association for Speech and Language Therapy (DBS e.V) (protocol code: 24‐TEMP547794‐KA‐ESpK and date of approval: 27‐06‐2024).

## Conflicts of Interest

The authors declare no conflicts of interest. The authors alone are responsible for the content and writing of the paper.

## Data Availability

The data that support the findings of this study are available from the corresponding author upon reasonable request.
